# Supplementary material for: SLAM-family receptors promote resolution of ILC2-mediated inflammation
Source: Nat Commun. 2024 Jun 13;15:5056. doi: 10.1038/s41467-024-49466-9 (PMC11176369; doi:10.1038/s41467-024-49466-9)
Supplement: Supplementary file 1 — Supplementary Information [file 41467_2024_49466_MOESM1_ESM.pdf]

Supplementary Information

SLAM-family receptors promote resolution of ILC2-mediated inflammation

Yuande Wang[1,2], Yuhe Quan[2], Junming He[2], Shasha Chen[1,3,4,5]* and Zhongjun Dong[1,2,3,4,5]*

1. Department of Allergy, the First Affiliated Hospital of Anhui Medical University and Institute of Clinical Immunology, Anhui Medical University, Hefei, 230032, China.
2. State Key Laboratory of Membrane Biology, School of Medicine and Institute for Immunology, Tsinghua University, Beijing, 100084, China.
3. Innovative Institute of Tumor Immunity and Medicine (ITIM), Hefei, 230032, China;
4. Anhui Province Key Laboratory of Tumor Immune Microenvironment and Immunotherapy, Hefei, 230032, China.
5. Inflammation and Immune Mediated Diseases Laboratory of Anhui Province, Anhui Medical University, Hefei, 230032, China

Correspondence to：

Dr. Zhongjun Dong, Medical Blvd. D328, Tsinghua University, Beijing, 100086, China; Phone: +86-10-62798536; dongzj@mail.tsinghua.edu.cn.

Dr. Shasha Chen, Zhixing Blvd. 1003, Anhui Medical University, Hefei, 230032, China; Cell phone: +86-18911821640; chenshasha.26@163.com

Supplemental Figures and Legends

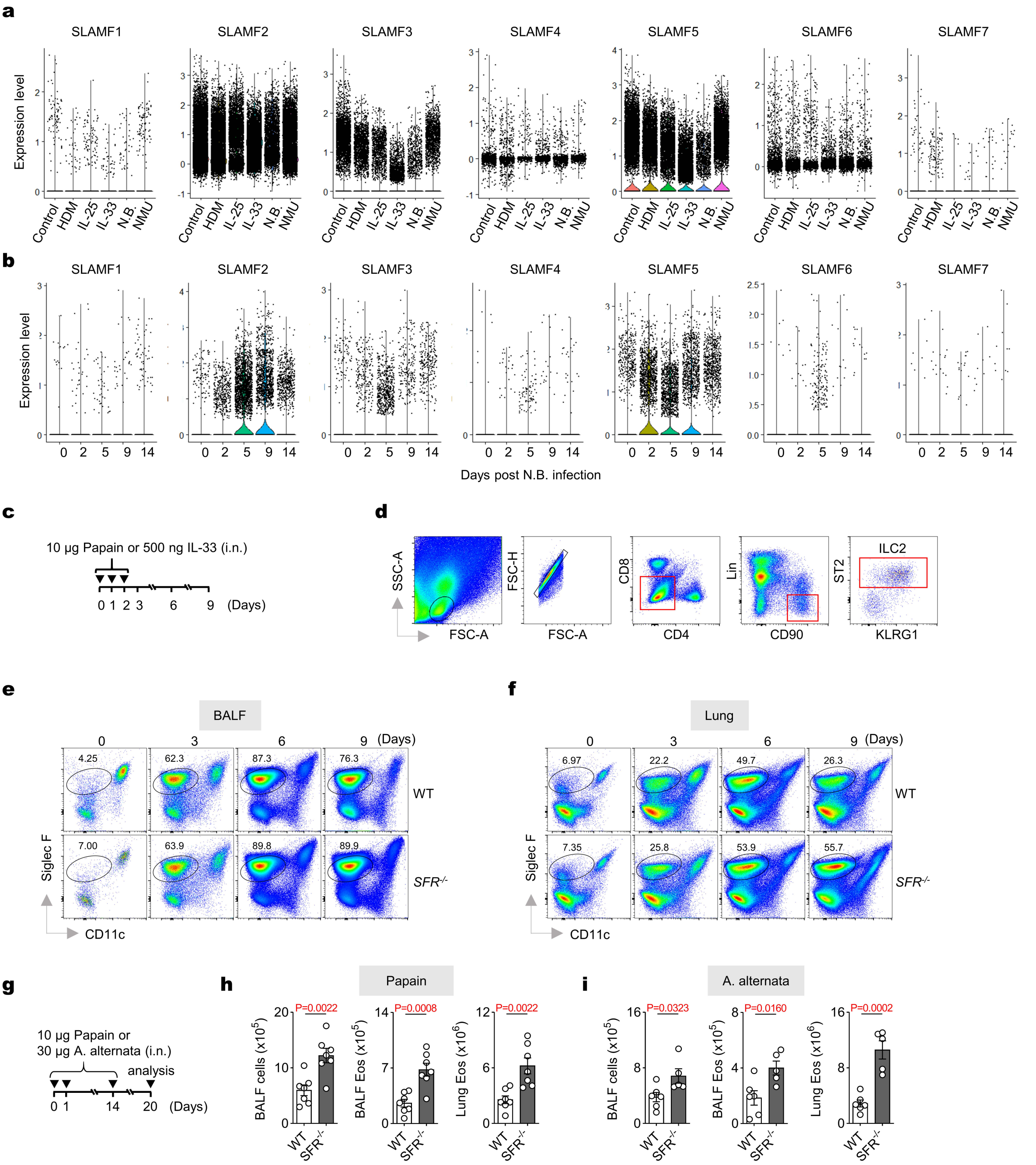

Supplementary Figure 1: related to Fig. 1.

a Gene expression analysis of ILC2s under the indicated treatment was conducted using the single-cell RNA datasets from the GEO database (GSE102299 and GSE131996). **b** Gene-expression analysis of ILC2s on the indicated days of Nippostrongylus brasiliensis treatment was conducted using the single-cell RNA dataset (GSE131996). **c** Mice received daily intranasal injections with papain or IL-33 on days 0, 1, and 2 were sacrificed for analysis on the day 0, 3, 6, or 9. **d** Flow cytometry detection of ILC2s (Lin^{-} CD90^{+}ST2^{+}). **e**, **f** Representative flow cytometric analysis of eosinophils (Siglec F^{+}CD11c^{-}) in BALF (**e**) and lungs (**f**) from WT and *SFR*$^{-/-}$ mice treated with papain (i.n., on day 0, 1, and 2) on the indicated days. **g** Mice received daily intranasal injections with papain or A. alternata on days 0, 1, and 14 and were sacrificed for analysis on day 20. **h**, **i** The number of BALF cells and eosinophils from WT and *SFR*$^{-/-}$ mice on day 20 after papain (**h**) or A. alternata (**i**) treatment (i.n., on day 0, 1, and 14). n=7 mice per group (**h**);

n(WT)=6 and n(*SFR*$^{-/-}$)=5 mice (**i**). The data (**h**, **i**) represent two independent experiments with similar results. All data are presented as means ± SEM, and statistical analysis was performed using two-tailed Student's t-test (**h**, **i**).

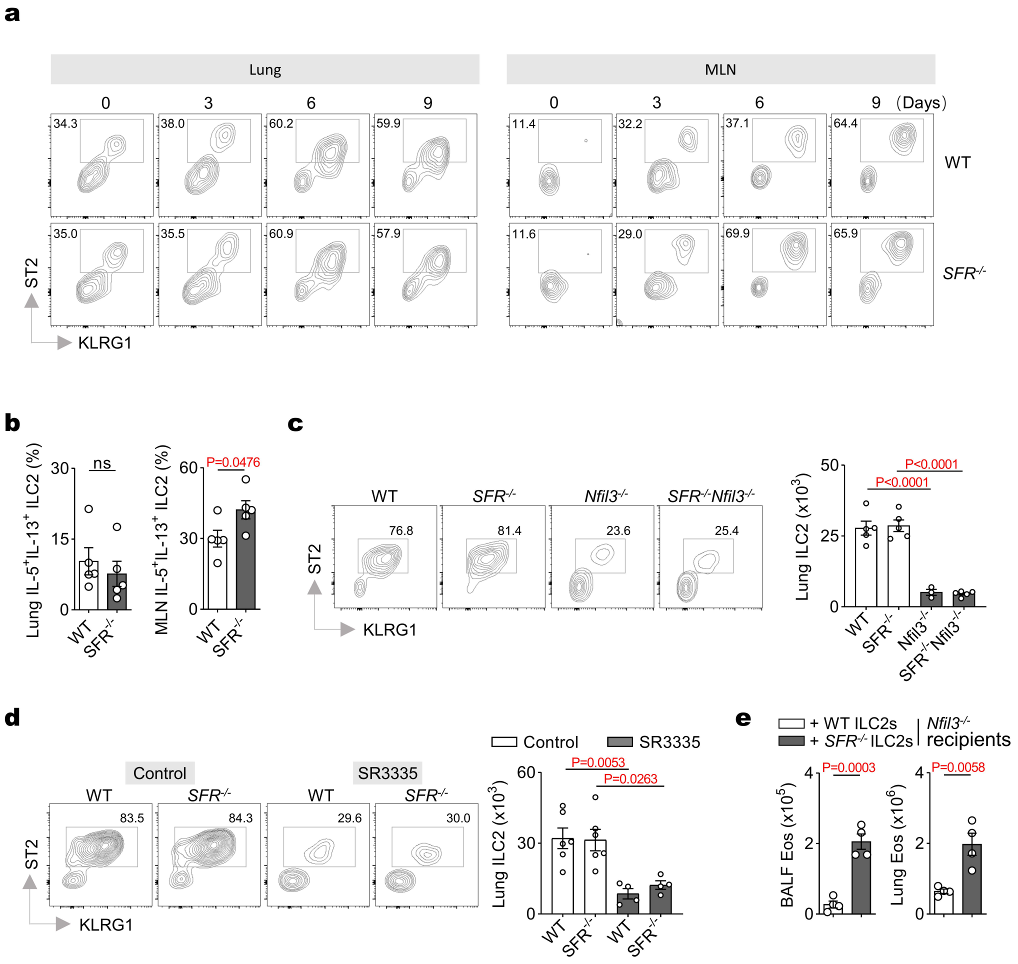

Supplementary Figure 2: related to Fig. 2.

a Representative flow cytometry detection of ILC2s (Lin^{-}CD90^{+}ST2^{+}, as illustrated in Supplementary Fig. 1d) in lung and MLN of mice at the indicated days following papain treatment (i.n., day 0, 1 and 2). **b** Quantification showing IL-5 and IL-13 expressing in gated lung and MLN ILC2s after 3 hours of PMA plus ionomycin restimulation on day 6 following A. alternata treatment (i.n., day 0, 1 and 2). n=5 mice per group. **c** Flow cytometric analysis and quantification of ILC2s in the lung of mice at day 9 following papain treatment (i.n., day 0, 1 and 2). n=5, 5, 3, 5 mice (left to right groups). **d** WT and *SFR*$^{-/-}$ mice were intraperitoneally treated with either SR3335 or DMSO (control). Flow cytometric analysis and quantification of ILC2s in the lung of mice at day 9 following papain treatment (i.n., day 0, 1 and 2). n=6, 6, 4, 4 mice (left to right groups). **e** ILC2s from WT and *SFR*$^{-/-}$ mice were intravenously injected into *Nfil3*$^{-/-}$ recipients 5 days prior to papain treatment (i.n., on days 0, 1, and 2), and the number of eosinophils was quantified on day 9. n=4 mice per group. The data represent at least two independent experiments with similar results. All data are represented as means ± SEM, and statistical analysis was performed using two-tailed Student's *t* test (**b**, **e**) or one-way ANOVA (**c**, **d**). ns, not significant.

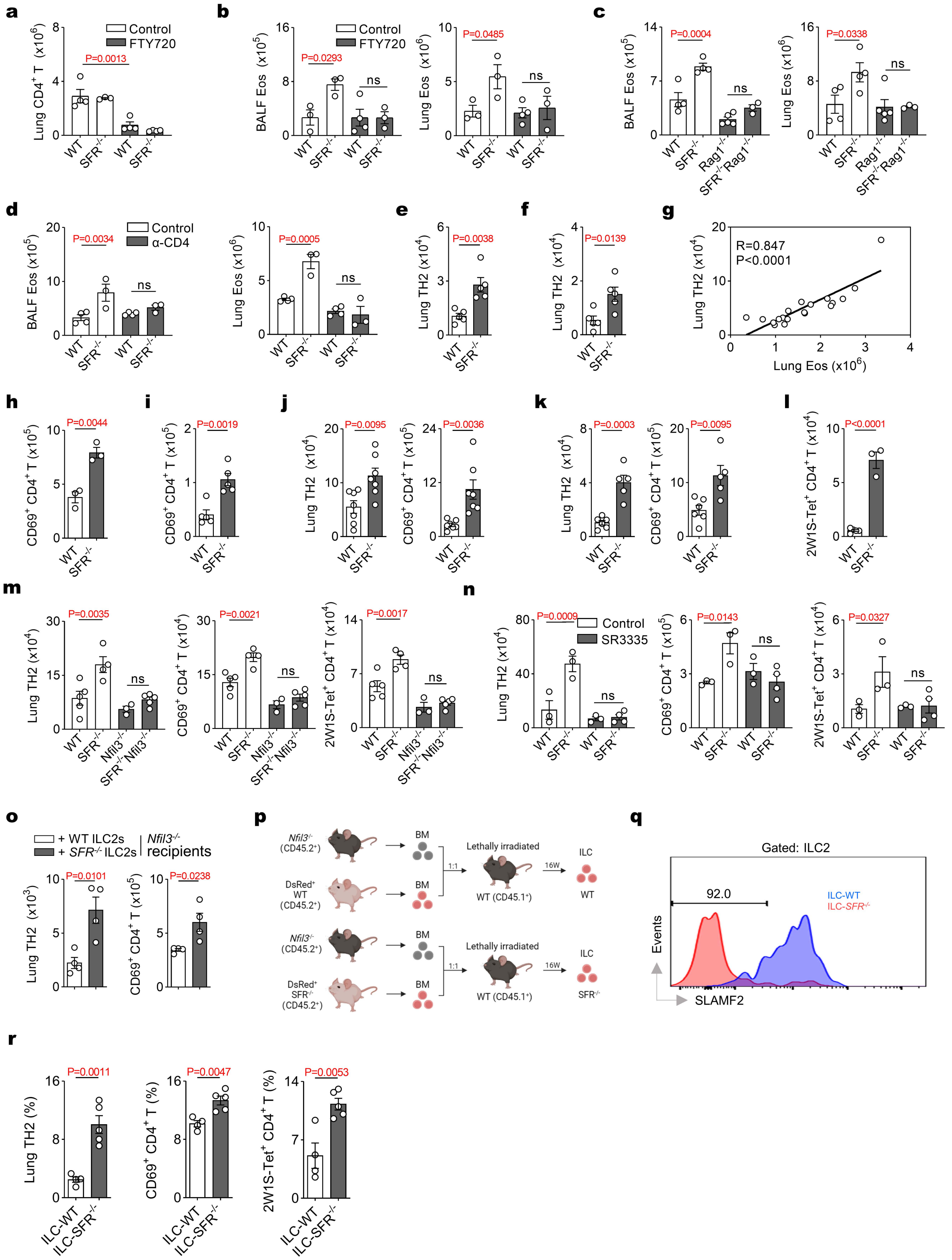

Supplementary Figure 3: related to Fig. 3.

a, **b** Detection of indicated cells in FTY720-treated mice at day 9 following IL-33 treatment (i.n., day 0/1/2). n=4/3/4/4 (**a**) and 3/3/4/3 (**b**) mice respectively. **c** Eosinophil detection in mice at day 9 following IL-33 treatment (i.n., day 0/1/2). n=4/4/5/3 mice respectively. **d** Eosinophil detection on day 9 in IL-33-induced (i.n., day 0/1/2) mice depleted of $CD4^+$ T cells. α-CD4, anti-CD4 antibody; control, IgG1. n=4/3/4/3 mice respectively. **e**, **f** TH2 cell detection in mice on day 9 following IL-33 (**e**) or A. alternata (**f**) treatment (i.n., day 0/1/2). n=5 mice per group. **g** The correlation analysis between the numbers of TH2 cells and eosinophils from mice at day 9 following IL-33 treatment (i.n., day 0/1/2). n=19. **h**, **i**

Detection of $CD69^+CD4^+$ T cells in mice on day 9 following IL-33 (**h**) or A. alternata (**i**) treatment (i.n., day 0/1/2). n=3 (**h**) or 5 (**i**) mice per group. **j**, **k** Detection of indicated cells in mice on day 20 following papain (**j**) or A. alternata (**k**) treatment (i.n., day 0/1/14). n=7 mice per group (**j**); n=6/5 mice respectively (**k**). **l**, **m** Detection of indicated cells in mice treated with peptide-2W1S+IL-33 (i.n., day 0/1/2) on day 9. n=5/3 (**l**) and 5/4/3/5 (**m**) mice respectively. **n** Detection of indicated cells in SR3335-treated mice at day 9 following peptide-2W1S+IL-33 treatment (i.n., day 0/1/2). n=3/3/3/4 mice respectively. **o** WT or *SFR*$^{-/-}$ ILC2s were intravenously injected into *Nfil3*$^{-/-}$ recipients five days prior to papain treatment (i.n., day 0/1/2), and the numbers of indicated cells in lungs were quantified on day 9. n=4 mice per group. **p**-**r** The generation of mixed bone-marrow chimera (**p**). Detection of SLAMF2 expression on ILC2s (**q**) and the proportion of $DsRed^-CD45.1^-CD45.2^+$ indicated cells (**r**) in recipients at day 9 following peptide-2W1S+IL-33 treatment (i.n., day 0/1/2). n(WT)=4 and n(*SFR*$^{-/-}$)=5. The data represent at least two independent experiments with similar results. All data are represented as means ± SEM, and statistical analysis was conducted using one-way ANOVA (**a**-**d**, **m**, **n**) or a two-tailed Student's t test (**e**, **f**, **h**-**l**, **o**, **r**) or linear regression (**g**). ns, not significant. Figure **p** created with BioRender.com released under a Creative Commons Attribution-NonCommercial-NoDerivs 4.0 International license.

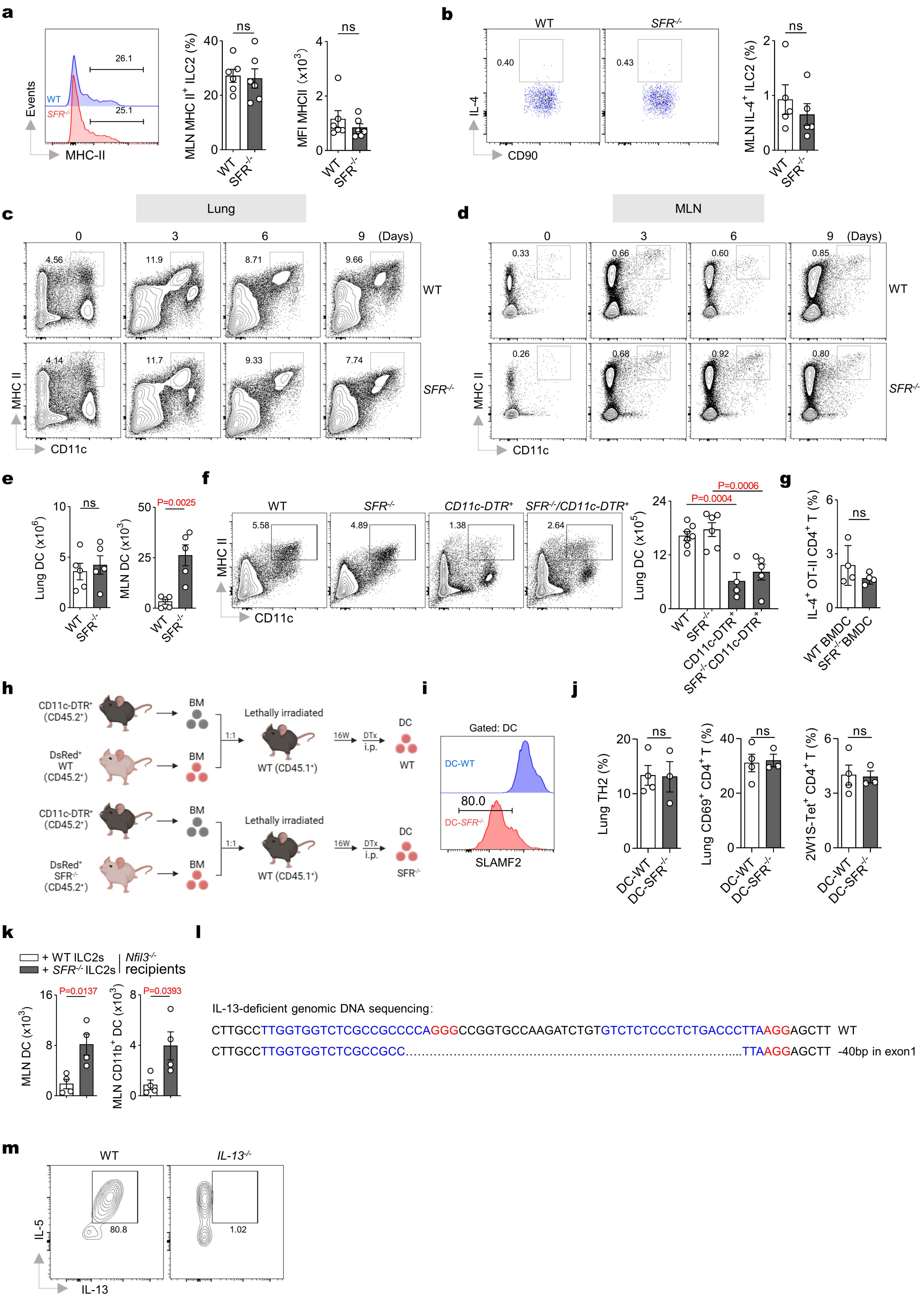

Supplementary Figure 4: related to Fig. 4.

a Detection of MHC II expression in MLN ILC2s of mice on day 6 after papain treatment (i.n., day 0/1/2). n=6 mice per group. **b** After 3 hours of PMA plus ionomycin restimulation on day 6, detection of IL-4 expression in MLN ILC2s of mice following papain treatment (i.n., day 0/1/2). n=5 mice per group. **c**, **d**

Representative flow cytometry of DCs in the lungs (**c**) and MLN (**d**) on the indicated days after papain treatment (i.n., day 0/1/2). **e** The number of DCs in lung and MLN from mice on day 6 after A. alternata treatment (i.n., day 0, 1 and 2). n=5 mice per group. **f** Quantification of lung DCs in DTx-treated mice at day 9 following papain treatment (i.n., day 0/1/2). n =7/6/4/5 mice respectively. **g** WT or *SFR*$^{-/-}$ BMDCs were co-cultured with WT OT-II^{+}CD4^{+} T cells in the presence of OVA323-339 peptide for 5 days, and the expression of IL-4 by WT OT-II^{+}CD4^{+} T cells was analyzed using intracellular staining after a 3-hour restimulation with PMA plus ionomycin. n=4. **h**-**j** Generation of mixed bone-marrow chimera (**h**, created with BioRender.com with permission). Detection of SLAMF2 expression on DCs (**i**) and the proportion of DsRed^{-}CD45.1^{-}CD45.2^{+} indicated cells (**j**) in recipients at day 9 following peptide-2W1S+IL-33 treatment (i.n., day 0/1/2). n(WT)=4 and n(*SFR*$^{-/-}$)=3. **k** ILC2s from WT and *SFR*$^{-/-}$ mice were intravenously injected into *Nfil3*$^{-/-}$ recipients five days prior to papain treatment (i.n., on days 0, 1, and 2), and the number of DCs in lungs and MLN was quantified on day 6. n=4 mice per group. **l** Generation of IL-13 knockout mice. The sgRNA sequence is labeled in blue; the protospacer-adjacent motif (PAM) sequence is labeled in red. **m** After 3 hours of PMA plus ionomycin restimulation on day 6, detection of IL-13 expression in MLN ILC2s of mice following papain treatment (i.n., day 0/1/2). The data (**a**-**k**, **m**) represent at least two independent experiments with similar results. All data are presented as means ± SEM, and statistical analysis was conducted using two-tailed Student's t test (**a**, **b**, **e**, **g**, **j**, **k**) or one-way ANOVA (**f**). ns, not significant. Figure **h** created with BioRender.com released under a Creative Commons Attribution-NonCommercial-NoDerivs 4.0 International license.

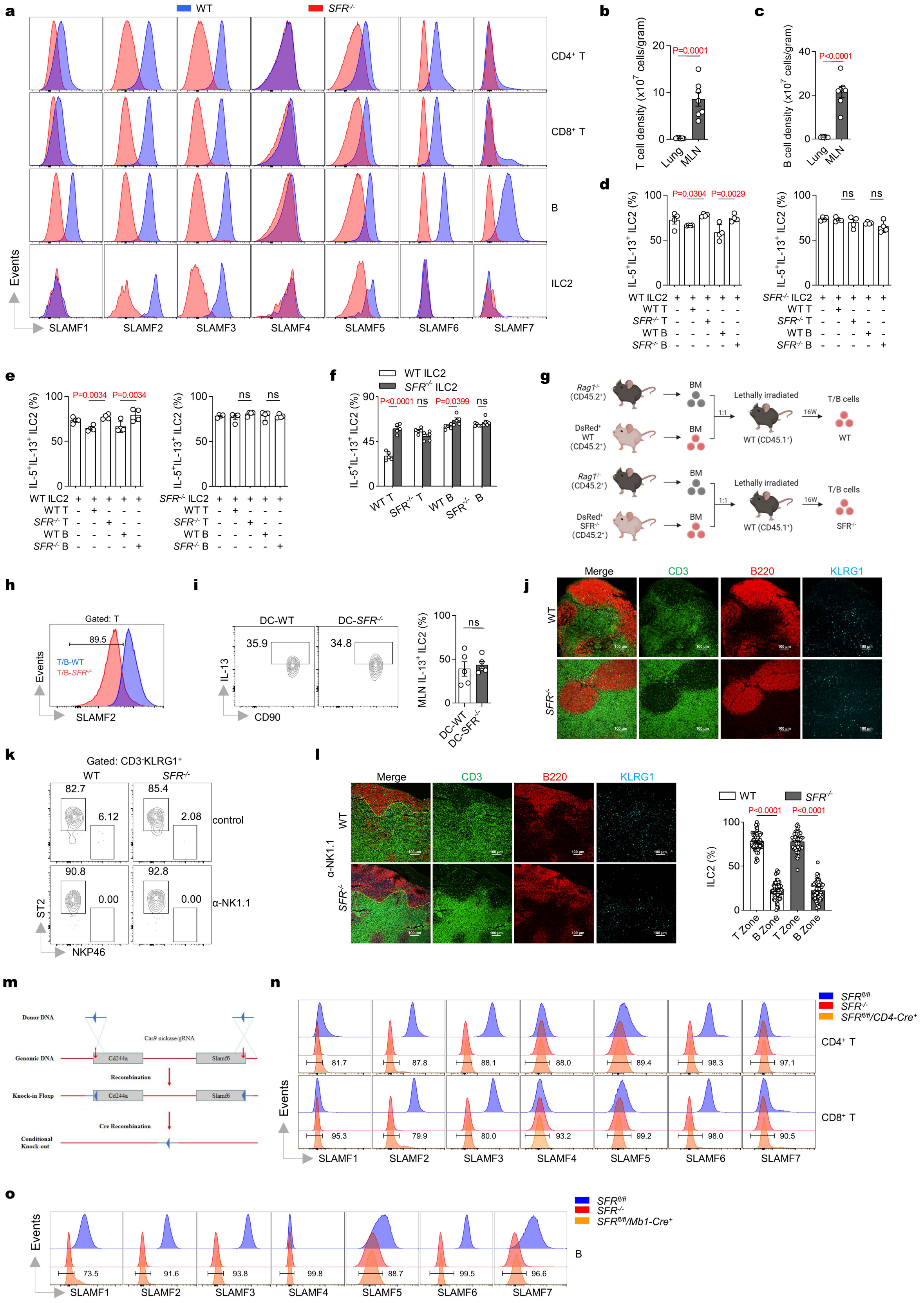

Supplementary Figure 5: related to Fig. 5.

a Detection of SFRs expression of indicated cells within MLN of mice on day 6 after papain treatment (i.n., day 0/1/2). **b**, **c** Quantification of T- (**b**) and B-cell (**c**) density in lungs and MLN of WT mice following 6 days post-papain treatment (i.n., day 0/1/2). n = 7 mice/group. **d**, **e** 1x10^4 (**d**) or 1x10^3 (**e**) WT or SFR-deficient ILC2s were stimulated with 1x10^5 (**d**) or 1x10^4 (**e**) WT or SFR-deficient T- or B-cells, and IL-5 and

IL-13 expression by ILC2s was analyzed. The cell counts for ILC2-only group are 1.1×10^5 (**d**) or 1×10^3 (**e**). n = 4. **f** An equal mixture of WT and SFR-deficient ILC2s (1×10^4) was co-cultured with either 1×10^5 WT or SFR-deficient T- or B-cells, and IL-5 and IL-13 expression by ILC2s was analyzed. n = 5. **g**, **h** The generation of mixed bone-marrow chimera (**g**). Detection of SLAMF2 expression on T cells (**h**). **i** After 3 hours of PMA plus ionomycin restimulation on day 6, detection of IL-13 expression in MLN $DsRed^+CD45.1^-CD45.2^+$ ILC2s of mice (as illustrated in Supplementary Fig. 4h) following papain treatment (i.n., day 0/1/2). n=5 mice/group. **j** Immunofluorescence staining of MLN ILC2s (related to Fig. 5f). **k** NK cell detection in the MLN on day 6 after papain treatment (i.n., on days 0, 1, and 2) in mice depleted of NK cells with α-NK1.1 or IgG1 control. **l** Immunofluorescence detection of MLN ILC2s on day 6 in the papain-induced (i.n., day 0, 1 and 2) mice depleted of NK cells. Sky blue sphere: KLRG1-positive and CD3-negative, representing ILC2s. n=73(WT) and 57($SFR^{-/-}$) field. **m** A strategy to generate $SFR^{fl/fl}$ mice. **n**, **o** Detection of SFRs expression in MLN T- (**n**) and B-cells (**o**) of mice on day 6 after IL-33 treatment (i.n., day 0, 1 and 2). The data are representative of at least two independent experiments with similar results. All data are presented as means ± SEM, and statistical analysis was conducted using a two-tailed Student's t test (**b**, **c**, **i**) or one-way ANOVA (**d**, **e**, **l**) or two-way ANOVA (**f**). ns, not significant. Figure **g** created with BioRender.com released under a Creative Commons Attribution-NonCommercial-NoDerivs 4.0 International license.

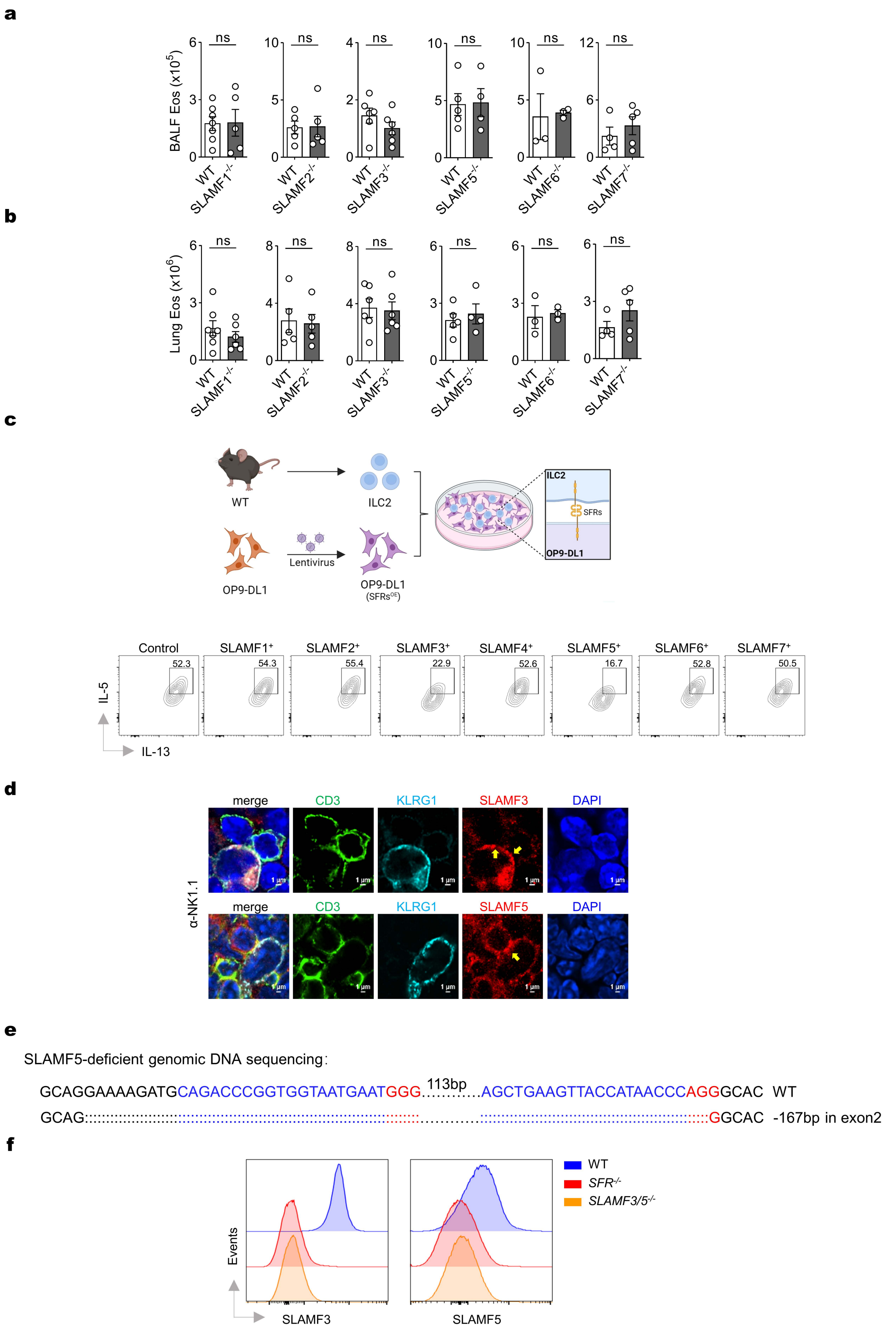

Supplementary Figure 6: related to Fig. 6.

a The number of eosinophils within BALF was quantified on day 9 after IL-33 treatment (i.n., day 0, 1 and 2). n=7, 5, 5, 5, 6, 6, 5, 4, 3, 3, 4, 5 mice (left to right groups). **b** The number of eosinophils within lungs was quantified on day 9 after IL-33 treatment (i.n., day 0, 1 and 2). n=7, 6, 5, 5, 6, 6, 5, 4, 3, 3, 4, 5 mice (left to right groups). **c** WT ILC2s were stimulated with OP9-DL1 cells ectopically expressing

individual SFR member in the presence of IL-7 plus IL-33 for 72 hours, resulting in the interaction between the ligands expressed on OP9-DL1 and the corresponding SFRs expressed on ILC2s (top panel). Representative intracellular staining demonstrates the expression of IL-5 and IL-13 by ILC2s following a 3-hour restimulation with PMA plus ionomycin (bottom panel). **d** Confocal microscopic analysis of the polarization of SLAMF3 or SLAMF5 (red) on the contact surface (yellow arrow) between ILC2s (KLRG1, sky blue) and their bystander T cells (CD3, green) within the MLN from NK cell-depleted mice on day 6 following IL-33 treatment (i.n., day 0, 1 and 2). **e** Generation of *SLAMF3/5*$^{-/-}$ mice. *SLAMF3/5*$^{-/-}$ mice were generated after CRISPR-Cas9-based genome editing SLAMF5 gene on *SLAMF3*$^{-/-}$ mice. The sgRNA sequence is labeled in blue; the PAM sequence is labeled in red. **f** Flow cytometry analysis was performed to assess the expression of SLAMF3 and SLAMF5 by ILC2s in the indicated mice. The data (**a**-**d**, **f**) are representative of at least two independent experiments with similar results. All data are presented as means ± SEM, and statistical analysis was conducted using a two-tailed Student's t test (**a**, **b**). ns, not significant. Figure **c** (top panel) created with BioRender.com released under a Creative Commons Attribution-NonCommercial-NoDerivs 4.0 International license.

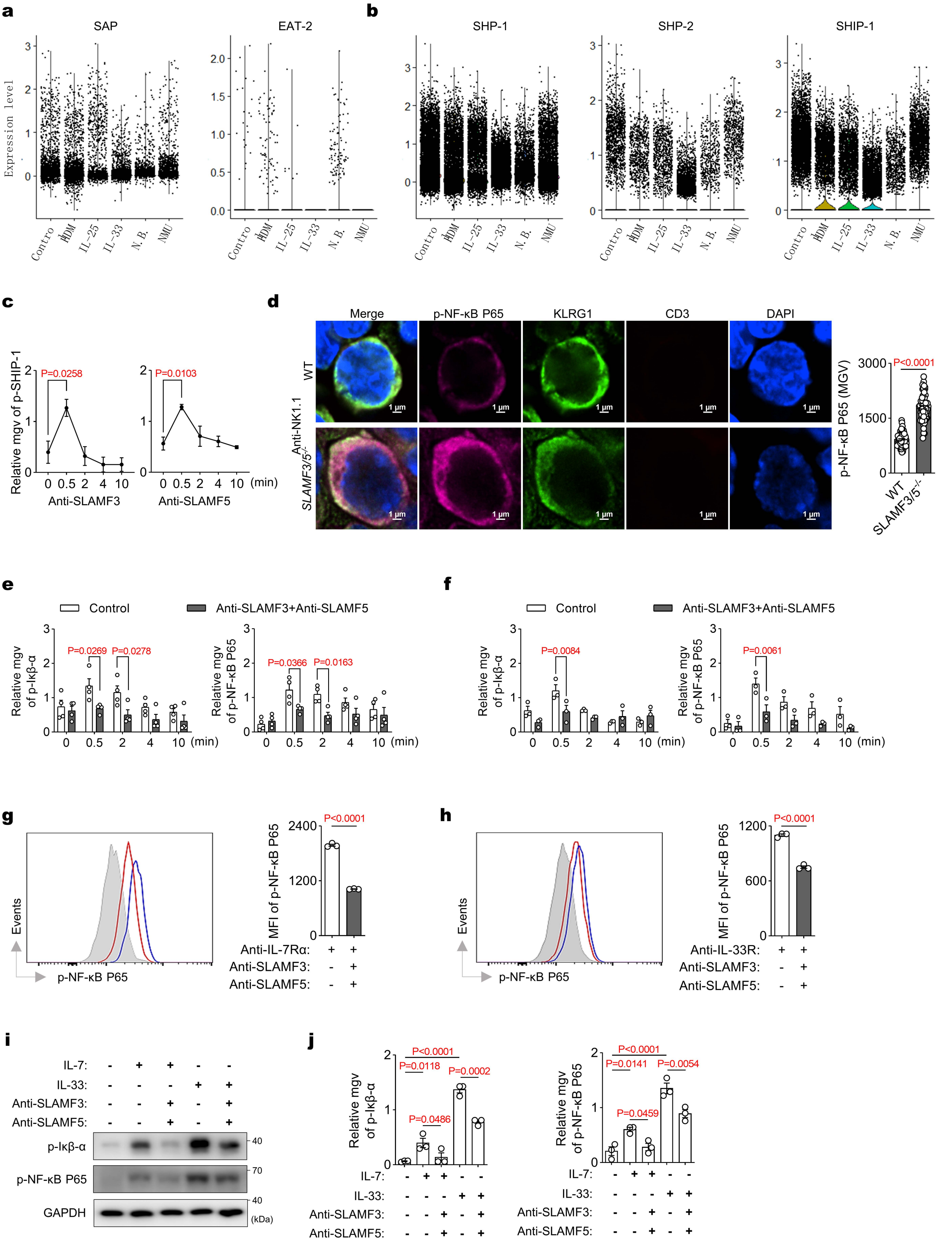

Supplementary Figure 7: related to Fig. 7.

a, **b** Gene expression analysis of ILC2s under the indicated treatment was conducted using the single-cell RNA datasets from the GEO database (GSE102299 and GSE131996). **c** Related to Fig. **7g**, the phosphorylation level of SHIP-1 was quantified. n=3 independent experiments. **d** Confocal imaging was performed to quantitatively visualize the phosphorylation of NF-κB (P-NF-κB P65, purple) in MLN ILC2s from the NK cell-depleted mice on day 6 following IL-33 treatment (i.n., day 0, 1, and 2). Representative

images (left panel) and quantification (right panel) are shown. n=81 (WT) or 61 (*SLAMF3/5*$^{-/-}$) cells per group. **e** Related to Fig. 7L, the phosphorylation level of Iκβ-α and NF-κB P65 was quantified. n=4 independent experiments. **f** Related to Fig. 7m, the phosphorylation level of Iκβ-α and NF-κB P65 was quantified. n=3 independent experiments. **g**, **h** Flow cytometry analysis was performed to assess the phosphorylation of NF-κB (p-NF-κB P65) in WT ILC2s stimulated with antibodies targeting IL-7Rα (Anti-IL-7Rα, **g**) or IL-33R (Anti-IL-33R, **h**), in the presence (+, red line) or absence (-, blue line) of antibodies against SLAMF3 and SLAMF5 (Anti-SLAMF3/5) for 2 minutes. Gray shade, representing isotype control. n=3 per group. **i**, **j** Western blot analysis was performed to assess NF-κB activation in WT ILC2s stimulated with (+) or without (-) IL-7 or IL-33, in the presence (+) or absence (-) of plate-bound anti-SLAMF3 and anti-SLAMF5 antibodies for 30 minutes (**i**). The phosphorylation level of Iκβ-α and NF-κB P65 was quantified (**j**). n=3 independent experiments. The data (**c**-**j**) are representative of two independent experiments with similar results. All data are presented as means ± SEM, and statistical analysis was conducted using one-way ANOVA (**c**, **j**), two-tailed Student's t test (**d**, **g**, **h**) or two-way ANOVA (**e**, **f**).

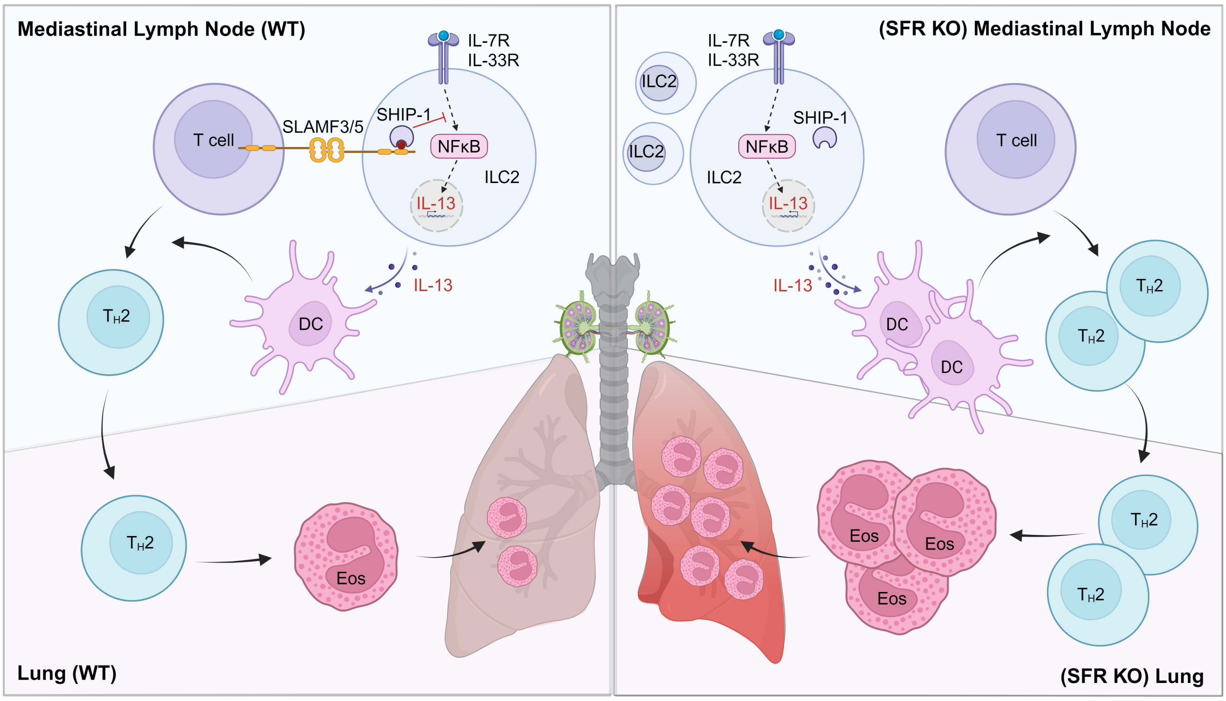

Supplementary Figure 8: graphical abstract.

(Left panel) T cells in the mediastinal lymph nodes (MLN) display increased interactions with neighboring ILC2s. The homotypic engagement of SLAMF3 and SLAMF5 (SLAMF3/5) between ILC2s and T cells, suppress the downstream NF-κB pathway of IL-7/IL-33 signaling through SHIP-1 phosphatase in ILC2s. This leads to decreased IL-13 production by ILC2s, contributing to a reduction in DC-driven TH2 response and ultimately aiding in the resolution of airway inflammation. (Right panel) Conversely, the absence of SLAM family receptors (SFR KO) results in heightened IL-13 production by MLN ILC2s, thereby amplifying the DC-driven TH2 response and aggravating pulmonary inflammation. This image created with BioRender.com released under a Creative Commons Attribution-NonCommercial-NoDerivs 4.0 International license
