# Supplementary material for: SLAM-family receptors promote resolution of ILC2-mediated inflammation
Source: Nat Commun. 2024 Jun 13;15:5056. doi: 10.1038/s41467-024-49466-9 (PMC11176369; doi:10.1038/s41467-024-49466-9)
Supplement: Supplementary file 3 — Reporting Summary [file 41467_2024_49466_MOESM3_ESM.pdf]

nature portfolio

Corresponding author(s): Shasha Chen, Zhongjun Dong

Last updated by author(s): Apr 29, 2024

Reporting Summary

Nature Portfolio wishes to improve the reproducibility of the work that we publish. This form provides structure for consistency and transparency in reporting. For further information on Nature Portfolio policies, see our Editorial Policies and the Editorial Policy Checklist.

Statistics

For all statistical analyses, confirm that the following items are present in the figure legend, table legend, main text, or Methods section.

n/a	Confirmed	
☐	☒	The exact sample size (*n*) for each experimental group/condition, given as a discrete number and unit of measurement
☐	☒	A statement on whether measurements were taken from distinct samples or whether the same sample was measured repeatedly
☐	☒	The statistical test(s) used AND whether they are one- or two-sided<br>*Only common tests should be described solely by name; describe more complex techniques in the Methods section.*
☐	☒	A description of all covariates tested
☐	☒	A description of any assumptions or corrections, such as tests of normality and adjustment for multiple comparisons
☐	☒	A full description of the statistical parameters including central tendency (e.g. means) or other basic estimates (e.g. regression coefficient) AND variation (e.g. standard deviation) or associated estimates of uncertainty (e.g. confidence intervals)
☐	☒	For null hypothesis testing, the test statistic (e.g. *F*, *t*, *r*) with confidence intervals, effect sizes, degrees of freedom and *P* value noted<br>*Give P values as exact values whenever suitable.*
☒	☐	For Bayesian analysis, information on the choice of priors and Markov chain Monte Carlo settings
☒	☐	For hierarchical and complex designs, identification of the appropriate level for tests and full reporting of outcomes
☒	☐	Estimates of effect sizes (e.g. Cohen's *d*, Pearson's *r*), indicating how they were calculated

Our web collection on statistics for biologists contains articles on many of the points above.

Software and code

Policy information about availability of computer code

Data collection	BD FACSDiva Software (version 8.0); Nis- elements 5.42. No custom software or code were used in the collection of data in this study.
Data analysis	GraphPad Prism (Version 8.0.2) ;FiJi (Version 1.54f); FlowJo (Version 10.5.3); STtudio (Version 20230601);R (Version 4.0.3);Nis- elements 5.42

For manuscripts utilizing custom algorithms or software that are central to the research but not yet described in published literature, software must be made available to editors and reviewers. We strongly encourage code deposition in a community repository (e.g. GitHub). See the Nature Portfolio guidelines for submitting code & software for further information.

Data

Policy information about availability of data

All manuscripts must include a data availability statement. This statement should provide the following information, where applicable:

- Accession codes, unique identifiers, or web links for publicly available datasets
- A description of any restrictions on data availability
- For clinical datasets or third party data, please ensure that the statement adheres to our policy

The previously published scRNA-seq data of GSE102299 (specifically GSM2733478 to GSM2733491) and GSE131996 (specifically GSM4192253 to GSM4192257) from GEO Gene Expression Omnibus (https://www.ncbi.nlm.nih.gov/geo/) database were used in this paper. The remaining data are available within the Article or Source Data file. Source data are provided with this paper.

Research involving human participants, their data, or biological material

Policy information about studies with human participants or human data. See also policy information about sex, gender (identity/presentation), and sexual orientation and race, ethnicity and racism.

Reporting on sex and gender	N/A
Reporting on race, ethnicity, or other socially relevant groupings	N/A
Population characteristics	N/A
Recruitment	N/A
Ethics oversight	N/A

Note that full information on the approval of the study protocol must also be provided in the manuscript.

Field-specific reporting

Please select the one below that is the best fit for your research. If you are not sure, read the appropriate sections before making your selection.

☒ Life sciences ☐ Behavioural & social sciences ☐ Ecological, evolutionary & environmental sciences

For a reference copy of the document with all sections, see nature.com/documents/nr-reporting-summary-flat.pdf

Life sciences study design

All studies must disclose on these points even when the disclosure is negative.

Sample size	No sample size calculation was performed. Sample size for animal experiment was chosen according to professional advice and experimental experience.
Data exclusions	No data were excluded.
Replication	Consistency of results was verified by repeated biological independent experiment at least two times. The number of replicates is clearly stated in the figure legends.
Randomization	Mice were randomly allocated into different groups.
Blinding	No blinding was used to assign and constitute experimental groups. Our readouts consist of objective measurements (such as flow cytometry) that do not require blinding for unbiased data analysis.

Behavioural & social sciences study design

All studies must disclose on these points even when the disclosure is negative.

Study description	*Briefly describe the study type including whether data are quantitative, qualitative, or mixed-methods (e.g. qualitative cross-sectional, quantitative experimental, mixed-methods case study).*
Research sample	*State the research sample (e.g. Harvard university undergraduates, villagers in rural India) and provide relevant demographic information (e.g. age, sex) and indicate whether the sample is representative. Provide a rationale for the study sample chosen. For studies involving existing datasets, please describe the dataset and source.*
Sampling strategy	*Describe the sampling procedure (e.g. random, snowball, stratified, convenience). Describe the statistical methods that were used to predetermine sample size OR if no sample-size calculation was performed, describe how sample sizes were chosen and provide a rationale for why these sample sizes are sufficient. For qualitative data, please indicate whether data saturation was considered, and what criteria were used to decide that no further sampling was needed.*
Data collection	*Provide details about the data collection procedure, including the instruments or devices used to record the data (e.g. pen and paper, computer, eye tracker, video or audio equipment) whether anyone was present besides the participant(s) and the researcher, and whether the researcher was blind to experimental condition and/or the study hypothesis during data collection.*
Timing	*Indicate the start and stop dates of data collection. If there is a gap between collection periods, state the dates for each sample cohort.*

Data exclusions	*If no data were excluded from the analyses, state so OR if data were excluded, provide the exact number of exclusions and the rationale behind them, indicating whether exclusion criteria were pre-established.*
Non-participation	*State how many participants dropped out/declined participation and the reason(s) given OR provide response rate OR state that no participants dropped out/declined participation.*
Randomization	*If participants were not allocated into experimental groups, state so OR describe how participants were allocated to groups, and if allocation was not random, describe how covariates were controlled.*

Ecological, evolutionary & environmental sciences study design

All studies must disclose on these points even when the disclosure is negative.

Study description	*Briefly describe the study. For quantitative data include treatment factors and interactions, design structure (e.g. factorial, nested, hierarchical), nature and number of experimental units and replicates.*
Research sample	*Describe the research sample (e.g. a group of tagged Passer domesticus, all Stenocereus thurberi within Organ Pipe Cactus National Monument), and provide a rationale for the sample choice. When relevant, describe the organism taxa, source, sex, age range and any manipulations. State what population the sample is meant to represent when applicable. For studies involving existing datasets, describe the data and its source.*
Sampling strategy	*Note the sampling procedure. Describe the statistical methods that were used to predetermine sample size OR if no sample-size calculation was performed, describe how sample sizes were chosen and provide a rationale for why these sample sizes are sufficient.*
Data collection	*Describe the data collection procedure, including who recorded the data and how.*
Timing and spatial scale	*Indicate the start and stop dates of data collection, noting the frequency and periodicity of sampling and providing a rationale for these choices. If there is a gap between collection periods, state the dates for each sample cohort. Specify the spatial scale from which the data are taken*
Data exclusions	*If no data were excluded from the analyses, state so OR if data were excluded, describe the exclusions and the rationale behind them, indicating whether exclusion criteria were pre-established.*
Reproducibility	*Describe the measures taken to verify the reproducibility of experimental findings. For each experiment, note whether any attempts to repeat the experiment failed OR state that all attempts to repeat the experiment were successful.*
Randomization	*Describe how samples/organisms/participants were allocated into groups. If allocation was not random, describe how covariates were controlled. If this is not relevant to your study, explain why.*
Blinding	*Describe the extent of blinding used during data acquisition and analysis. If blinding was not possible, describe why OR explain why blinding was not relevant to your study.*

Did the study involve field work? ☐ Yes ☐ No

Field work, collection and transport

Field conditions	*Describe the study conditions for field work, providing relevant parameters (e.g. temperature, rainfall).*
Location	*State the location of the sampling or experiment, providing relevant parameters (e.g. latitude and longitude, elevation, water depth).*
Access & import/export	*Describe the efforts you have made to access habitats and to collect and import/export your samples in a responsible manner and in compliance with local, national and international laws, noting any permits that were obtained (give the name of the issuing authority, the date of issue, and any identifying information).*
Disturbance	*Describe any disturbance caused by the study and how it was minimized.*

Reporting for specific materials, systems and methods

We require information from authors about some types of materials, experimental systems and methods used in many studies. Here, indicate whether each material, system or method listed is relevant to your study. If you are not sure if a list item applies to your research, read the appropriate section before selecting a response.

Materials & experimental systems

n/a	Involved in the study
☐	☒ Antibodies
☐	☒ Eukaryotic cell lines
☒	☐ Palaeontology and archaeology
☐	☒ Animals and other organisms
☒	☐ Clinical data
☒	☐ Dual use research of concern
☒	☐ Plants

Methods

n/a	Involved in the study
☒	☐ ChIP-seq
☐	☒ Flow cytometry
☒	☐ MRI-based neuroimaging

Antibodies

Antibodies used

Flow cytometry antibodies:
CD3e 145-2C11 PE-eFluor™610 Thermo 61-0031-82 1:500
CD3e 145-2C11 FITC Thermo 11-0031-82 1:500
CD19 eBio1D3 PE-eFluor™610 Thermo 61-0193-82 1:500
NK1.1 PK136 PE-eFluor™610 Thermo 61-5941-82 1:500
CD11b M1/70 PE-eFluor™610 Thermo 61-0112-82 1:500
Gr1 RB6-8C5 PE-eFluor™610 Thermo 61-5931-82 1:500
MHC-II M5/114.15.2 eFluor™450 Thermo 48-5321-82 1:500
CD11c N418 PE Thermo 12-0114-82 1:500
CD4 GK1.5 Alexa Fluor™700 Thermo 56-0041-82 1:500
CD8 53-6.7 PE-Cyanine7 Thermo 25-0081-82 1:500
ST2 RMST2-2 PE-Cyanine7 Thermo 25-9335-82 1:500
KLRG1 2F1 PerCP-eFluor™710 Thermo 46-5893-82 1:500
KLRG1 2F1 APC Thermo 17-5893-82 1:500
Siglec F 1RNM44N PerCP-eFluor™710 Thermo 46-1702-82 1:500
SLAMF2 HM48-1 APC Thermo 17-0481-82 1:500
SLAMF4 eBio244F4 APC Thermo 17-2441-82 1:500
CD45.1 A20 APC-eFluor™780 Thermo 47-0453-82 1:500
CD45.2 104 Brilliant Violet™605 Thermo 406-0454-82 1:500
CD90.2 53-2.1 eFluor™450 Thermo 48-0902-82 1:500
CD90.2 53-2.1 Super Bright™702 Thermo 67-0902-82 1:500
CD90.1 HIS51 FITC Thermo 11-0900-81 1:500
IL-4 11B11 APC Thermo 17-7041-82 1:250
IL-5 TRFK5 PE Thermo 12-7052-82 1:250
IL-13 eBio13A PerCP-eFluor™710 Thermo 46-7133-82 1:250
GATA-3 TWAJ Alexa Fluor™488 Thermo 53-9966-42 1:100
CD69 H1.2F3 eFluor™450 Thermo 48-0691-82 1:500
CD44 IM7 PE-Cyanine7 Thermo 25-0441-82 1:500
SLAMF1 TC15-12F12.2 APC BioLegend 115910 1:500
SLAMF3 ly9ab3 Biotin BioLegend 122903 1:500
SLAMF5 mCD84.7 PE BioLegend 122806 1:500
SLAMF6 330-AJ APC BioLegend 134610 1:500
SLAMF7 4G2 PE BioLegend 152006 1:500
SAP 1A9 PE BD 566729 1:200
IκBα Antibody L35A5 CST 4818 1:200
Phospho-SHIP1 (Tyr1020) Antibody CST 3941 1:200
Phospho-SHP-1 (Tyr564) (D11G5) Antibody CST 8849 1:200
Phospho-SHP-2 (Tyr542) (E8D6V) Antibody CST 15543 1:200
SHP-1 (E1U6R) Antibody CST 26516 1:200
SHP-2 (D50F2) Antibody CST 3397 1:200
SHIP1 (E8M5D) Antibody CST 9877 1:200
PE labeled tetramer of I-A(b) mouse 2W1S EAWGALANWAVDSA NIH 1:250
APC labeled tetramer of I-A(b) chicken ova 325-335 QAVHAAHAEIN NIH 1:250
Rabbit IgG (H+L) Secondary Antibody Alexa Fluor™647 Thermo A21244 1:500
Mouse IgG (H+L) Secondary Antibody FITC Thermo A16067 1:500
Streptavidin PE Thermo S21388 1:500

Western blot antibodies:
Phospho-SHIP1 (Tyr1020) Antibody CST 3941 1:1000
SHIP1 (E8M5D) Antibody CST 9877 1:1000
Phospho-NF-κB p65 (Ser536) (93H1) Antibody CST 3033 1:1000
Phospho-IκBα (Ser32/36) (5A5) Antibody CST 9246 1:1000
GAPDH (D16H11) Antibody CST 5174 1:1000

Immunofluorescence antibodies:
KLRG1 2F1 Functional Grade Thermo 16-5893-82 1:200
CD3 17A2 Functional Grade Thermo 16-0032-82 1:200
B220 RA3-6B2 PE Thermo 12-0452-82 1:200

SLAMF3 ly9ab3 Biotin BioLegend 122903 1:200
SLAMF5 mCD84.7 Biotin BioLegend 122803 1:200
Phospho-NF-κB p65 (Ser536) (93H1) Antibody CST 3033 1:200
Syrian Hamster IgG (H+L) Alexa Fluor™647 Thermo A21451 1:200
Rat IgG (H+L) Alexa Fluor™488 Thermo A11006 1:200
Rabbit IgG (H+L) Alexa Fluor™568 Thermo A11011 1:200
Streptavidin PE Thermo S21388 1:200

Validation

Antibodies were all sourced commercially with independent validations.

Eukaryotic cell lines

Policy information about cell lines and Sex and Gender in Research

Cell line source(s)

HEK-293T cells and OP9 stromal cells were from American Type Culture Collection

Authentication

the suppliers routinely authentiate the cell lines

Mycoplasma contamination

All cell lines tested for negative mycoplasma contamination.

Commonly misidentified lines
(See ICLAC register)

No commonly misidentified cell lines were used in the study.

Palaeontology and Archaeology

Specimen provenance

Provide provenance information for specimens and describe permits that were obtained for the work (including the name of the issuing authority, the date of issue, and any identifying information). Permits should encompass collection and, where applicable, export.

Specimen deposition

Indicate where the specimens have been deposited to permit free access by other researchers.

Dating methods

If new dates are provided, describe how they were obtained (e.g. collection, storage, sample pretreatment and measurement), where they were obtained (i.e. lab name), the calibration program and the protocol for quality assurance OR state that no new dates are provided.

☐ Tick this box to confirm that the raw and calibrated dates are available in the paper or in Supplementary Information.

Ethics oversight

Identify the organization(s) that approved or provided guidance on the study protocol, OR state that no ethical approval or guidance was required and explain why not.

Note that full information on the approval of the study protocol must also be provided in the manuscript.

Animals and other research organisms

Policy information about studies involving animals; ARRIVE guidelines recommended for reporting animal research, and Sex and Gender in Research

Laboratory animals

Mice lacking SLAM family members (SFR-/-), SLAMF1-deficient (SLAMF1-/-), SLAMF2-deficient (SLAMF2-/-), SLAMF3-deficient (SLAMF3-/-), SLAMF5-deficient (SLAMF5-/-), SLAMF6-deficient (SLAMF6-/-), SLAMF7-deficient (SLAMF7-/-), SLAMF3 and SLAMF5-double deficient (SLAMF3/5-/-), IL-13-deficient (IL-13-/-), SAP and EAT-2-double deficient (SAP-/-EAT-2-/-) mice were generated using CRISPR-Cas9-based genome editing in our lab(Chen et al., 2016; Li et al., 2022). CD11c-DTR mice were a gift from Zhihua Liu (Tsinghua University, Beijing, China). OT-II transgenic, Rag1-/-, CD45.1, and C57BL/6 mice were obtained from the Jackson Laboratory (Bar Harbor, Maine, USA). All mice were bred on a C57BL/6 background and maintained in specific pathogen-free animal facilities at Tsinghua University. All procedures involving animals were approved by the Animal Ethics Committee of Tsinghua University.The dark/light cycle is 12 hours/12 hours in the 24-hour cycle. The ambient temperature is 23-26℃, and humidity is 40%-70%.

Wild animals

No wild animals were used in the study.

Reporting on sex

The sex of mice was not considered in study design.

Field-collected samples

The study did not involve samples collected from the field.

Ethics oversight

All experiments using mice were approved by the Animal Ethics Committee of Tsinghua University.

Note that full information on the approval of the study protocol must also be provided in the manuscript.

Clinical data

Policy information about clinical studies

All manuscripts should comply with the ICMJE guidelines for publication of clinical research and a completed CONSORT checklist must be included with all submissions.

Clinical trial registration	*Provide the trial registration number from ClinicalTrials.gov or an equivalent agency.*
Study protocol	*Note where the full trial protocol can be accessed OR if not available, explain why.*
Data collection	*Describe the settings and locales of data collection, noting the time periods of recruitment and data collection.*
Outcomes	*Describe how you pre-defined primary and secondary outcome measures and how you assessed these measures.*

Dual use research of concern

Policy information about dual use research of concern

Hazards

Could the accidental, deliberate or reckless misuse of agents or technologies generated in the work, or the application of information presented in the manuscript, pose a threat to:

No	Yes	
☐	☐	Public health
☐	☐	National security
☐	☐	Crops and/or livestock
☐	☐	Ecosystems
☐	☐	Any other significant area

Experiments of concern

Does the work involve any of these experiments of concern:

No	Yes	
☐	☐	Demonstrate how to render a vaccine ineffective
☐	☐	Confer resistance to therapeutically useful antibiotics or antiviral agents
☐	☐	Enhance the virulence of a pathogen or render a nonpathogen virulent
☐	☐	Increase transmissibility of a pathogen
☐	☐	Alter the host range of a pathogen
☐	☐	Enable evasion of diagnostic/detection modalities
☐	☐	Enable the weaponization of a biological agent or toxin
☐	☐	Any other potentially harmful combination of experiments and agents

Plants

Seed stocks	N/A
Novel plant genotypes	N/A
Authentication	N/A

ChIP-seq

Data deposition

☐ Confirm that both raw and final processed data have been deposited in a public database such as GEO.

☐ Confirm that you have deposited or provided access to graph files (e.g. BED files) for the called peaks.

Data access links<br>*May remain private before publication.*	*For "Initial submission" or "Revised version" documents, provide reviewer access links. For your "Final submission" document, provide a link to the deposited data.*
Files in database submission	*Provide a list of all files available in the database submission.*
Genome browser session<br>(e.g. UCSC)	*Provide a link to an anonymized genome browser session for "Initial submission" and "Revised version" documents only, to enable peer review. Write "no longer applicable" for "Final submission" documents.*

Methodology

Replicates	*Describe the experimental replicates, specifying number, type and replicate agreement.*
Sequencing depth	*Describe the sequencing depth for each experiment, providing the total number of reads, uniquely mapped reads, length of reads and whether they were paired- or single-end.*
Antibodies	*Describe the antibodies used for the ChIP-seq experiments; as applicable, provide supplier name, catalog number, clone name, and lot number.*
Peak calling parameters	*Specify the command line program and parameters used for read mapping and peak calling, including the ChIP, control and index files used.*
Data quality	*Describe the methods used to ensure data quality in full detail, including how many peaks are at FDR 5% and above 5-fold enrichment.*
Software	*Describe the software used to collect and analyze the ChIP-seq data. For custom code that has been deposited into a community repository, provide accession details.*

Flow Cytometry

Plots

Confirm that:

☒ The axis labels state the marker and fluorochrome used (e.g. CD4-FITC).

☒ The axis scales are clearly visible. Include numbers along axes only for bottom left plot of group (a 'group' is an analysis of identical markers).

☒ All plots are contour plots with outliers or pseudocolor plots.

☒ A numerical value for number of cells or percentage (with statistics) is provided.

Methodology

Sample preparation	Sample preparation was described in "Methods" section.
Instrument	BD LSR II (four-laserBlue/Red/Violet/ultraviolet flow cytometry analyzer, BD Biosciences)
Software	BD FACSDiva (version 8.0); FlowJo (Version 10.5.3)
Cell population abundance	Approximately 90-95%
Gating strategy	Gating strategies were described in supplementary Figure S1.

☒ Tick this box to confirm that a figure exemplifying the gating strategy is provided in the Supplementary Information.

Magnetic resonance imaging

Experimental design

Design type	N/A
Design specifications	N/A

Behavioral performance measures: N/A

Acquisition

Imaging type(s): N/A

Field strength: N/A

Sequence & imaging parameters: N/A

Area of acquisition: N/A

Diffusion MRI ☐ Used ☒ Not used

Preprocessing

Preprocessing software: N/A

Normalization: N/A

Normalization template: N/A

Noise and artifact removal: N/A

Volume censoring: N/A

Statistical modeling & inference

Model type and settings: N/A

Effect(s) tested: N/A

Specify type of analysis: ☐ Whole brain ☐ ROI-based ☐ Both

Statistic type for inference (See Eklund et al. 2016): N/A

Correction: N/A

Models & analysis

n/a	Involved in the study
☒	☐ Functional and/or effective connectivity
☒	☐ Graph analysis
☒	☐ Multivariate modeling or predictive analysis

Functional and/or effective connectivity: *Report the measures of dependence used and the model details (e.g. Pearson correlation, partial correlation, mutual information).*

Graph analysis: *Report the dependent variable and connectivity measure, specifying weighted graph or binarized graph, subject- or group-level, and the global and/or node summaries used (e.g. clustering coefficient, efficiency, etc.).*

Multivariate modeling and predictive analysis: *Specify independent variables, features extraction and dimension reduction, model, training and evaluation metrics.*
